# Inhibitory Control Modulates the Orthographic Neighbourhood Frequency Effect in Chinese Two-Character Word Recognition: An ERP Study

**DOI:** 10.3390/bs16101918

**Published:** 2026-10-16

**Authors:** Jianping Xiong, Xiyue Zhang, Ping Ju, Weipeng Wang, Ming Zhang

**Affiliations:** 1 Faculty of Education, Henan Normal University, Xinxiang 453000, China; 2410183021@stu.htu.edu.cn (X.Z.); 2310183024@stu.htu.edu.cn (W.W.); zhangming@stu.htu.edu.cn (M.Z.); 2 Department of Psychology, Renmin University of China, Beijing 100000, China; 2010183022@stu.htu.edu.cn

**Keywords:** orthographic neighbourhood frequency, inhibitory control, lexical decision, event-related potentials, mixed design

## Abstract

In Chinese reading, inhibitory control has been proposed to influence the orthographic neighbourhood frequency (ONF) effect by regulating the activation of competing orthographic neighbours. However, it remains unclear whether individual differences in inhibitory control modulate the ONF effect during Chinese word recognition. In the present study, 36 participants with high inhibitory control (HIC) and 36 with low inhibitory control (LIC) were selected through a preliminary experiment. The formal experiment employed a 2 (inhibitory control: high vs. low) × 2 (ONF: high vs. low) mixed design, combining a lexical decision task with event-related potential (ERP) measures to investigate the influence of inhibitory control on the ONF effect in Chinese two-character word recognition. The main findings were as follows: (1) Behaviourally, reaction times showed significant main effects of ONF and inhibitory-control group, but the ONF × group interaction was not significant. For accuracy, only the main effect of group was significant; neither the main effect of ONF nor the ONF × group interaction reached significance. (2) ERP results showed ONF effects in both the N400 and LPC time windows. In the N400 component, a significant interaction between ONF and group was observed over the left posterior region. The LIC group elicited a larger amplitude in the condition with higher-frequency orthographic neighbours, whereas the HIC group exhibited comparable responses between the two ONF conditions. Taken together, the findings confirmed an inhibitory ONF effect in Chinese two-character word recognition. Although no ONF × inhibitory-control interaction was observed in behavioural performance, the significant interaction in the left posterior N400 suggests that individual differences in inhibitory control are reflected in ONF-related lexical-semantic processing at the neural level. These findings highlight the role of individual differences in inhibitory control in Chinese word recognition and suggest that readers may differ in their susceptibility to interference from higher-frequency orthographic neighbours.

## 1. Introduction

### 1.1. Orthographic Neighbourhood Frequency in Chinese Word Recognition

A central issue in psycholinguistics concerns how lexical information is represented in and retrieved from the mental lexicon. Previous studies have shown that orthographic neighbourhood frequency (ONF) is an important factor influencing lexical processing (Andrews, 1989). ONF refers to the frequency relationship between a target word and its orthographic neighbours. In studies of Chinese two-character word recognition, orthographic neighbours have commonly been operationalised as words that share a constituent character, particularly the initial character, with the target word (Huang et al., 2006; Li et al., 2015, 2017; Xiong et al., 2021). Recent megastudy evidence has further suggested that character-sharing neighbourhood effects in Chinese may partly reflect morphological-family structure, because individual Chinese characters also function as morphemes (Tsang et al., 2024). The ONF effect arises when a target word has one or more orthographic neighbours that occur more frequently than the target itself. Otherwise, the target has no higher-frequency neighbour (Huang et al., 2006; Li et al., 2017). For example, the target word 奇妙 (qímiào, “wonderful”) has initial-character neighbours such as 奇特 (“peculiar”) and 奇怪 (“strange”), both of which occur more frequently than the target, and therefore represents a target with higher-frequency orthographic neighbours. These higher-frequency neighbours can then affect how the target word is processed and recognised.

In Chinese reading, two-character words are related to their orthographic neighbours not only in form but also in meaning (Li et al., 2015). Processing a two-character word may also automatically activate the semantic representations of its orthographic neighbours. Thus, the ONF effect in Chinese may involve not only orthographic processing but also the semantic activation and processing of orthographic neighbours. Consistently with this view, recent behavioural and computational evidence indicates that constituent meanings are routinely combined during Chinese compound-word recognition, even in lexical decision tasks that do not explicitly require semantic analysis (Hsieh et al., 2025).

The Controlled Semantic Cognition framework (CSC; Lambon Ralph et al., 2017) proposes that successful semantic processing depends on the interaction between stored semantic representations and control processes that regulate the retrieval and selection of task-relevant information. Within this framework, inhibitory control, a core component of cognitive control (Diamond, 2013), may be particularly important when multiple lexical-semantic representations are activated simultaneously. Christopher et al. (2012) further proposed that, during reading, individuals need to continually engage inhibitory control to suppress irrelevant lexical activation during reading, retrieve the correct target word, and generate an appropriate response, thereby avoiding erroneous lexical guesses (i.e., identifying an orthographic neighbour in place of the target word) or incorrect matching and ultimately achieving successful recognition of the specific word. On this basis, inhibitory control may influence the ONF effect in Chinese two-character word recognition; however, this question remains largely unexplored in the existing literature. We use ERP measures to examine differences between individuals with HIC and LIC when processing target words with versus without higher-frequency orthographic neighbours, thereby providing empirical evidence for the above proposal.

### 1.2. Theoretical Framework: Inhibitory Control and Lexical-Semantic Competition

Accumulating evidence indicates that, in alphabetic languages, the ONF effect varies with orthographic transparency. Orthographic transparency refers to the consistency between spelling and phonology, that is, the extent to which pronunciation can be derived from orthographic form (Xie et al., 2022). Studies in French, Dutch, and Spanish have generally reported inhibitory effects of higher-frequency orthographic neighbours on target-word recognition (Grainger & Segui, 1990; Grainger & Ferrand, 1994; Grainger & Jacobs, 1996; Carreiras et al., 1997). Conversely, findings from less transparent languages, such as English, have been inconsistent. Some studies have reported facilitatory effects of higher-frequency orthographic neighbours on target-word recognition (Sears et al., 1995; Slattery, 2009; Camblats et al., 2022), whereas others have found inhibitory effects of higher-frequency neighbours (Pollatsek et al., 1999; Paap et al., 2000; Sears et al., 2006). Still other studies have found no ONF effect in word recognition (Forster & Shen, 1996; Huntsman & Lima, 2002).

Existing studies of the ONF effect in Chinese two-character word recognition have consistently shown that higher-frequency neighbours inhibit target-word recognition (Huang et al., 2006; Li et al., 2017). More recent sentence-reading evidence indicates that this inhibitory effect is sensitive to contextual predictability: the higher-frequency-neighbour effect was observed for unpredictable target words but disappeared when the target word was highly predictable from the preceding sentence context (Yao et al., 2022). In addition, inhibitory neighbourhood-frequency effects have recently been reported in Chinese character recognition under both stroke-based and radical-based definitions of orthographic neighbours (Y. Liu et al., 2023). More recent research on orthographic neighbourhood size has also found a facilitatory effect during Chinese two-character word recognition, with semantic activation playing an important role in this effect (Xiong et al., 2026). Taken together, orthographic competition alone may not fully account for the ONF effect in Chinese. Semantic-level activation and inhibition may also contribute to this effect.

Previous models have generally attributed the inhibitory effect of orthographic neighbourhood frequency to automatic lexical activation and competition. During word recognition, multiple lexical candidates may become activated and compete with one another before the target word is selected. A classic example is the Interactive Activation Model (IAM; McClelland & Rumelhart, 1981). Within this framework, word recognition is achieved through bidirectional activation between visual features, letters, and words. At the word level, lexical representations inhibit one another through lateral inhibition, resulting in competition among lexical candidates. During target-word recognition, higher-frequency orthographic neighbours intensify within-level competition and inhibit lower-frequency target words.

Grainger and Jacobs (1996) extended this account by introducing the Multiple Read-Out Model (MROM), which posits that word recognition relies on three parallel decision criteria: the M criterion (single-word activation), the Σ criterion (global activation), and the T criterion (time deadline). When a target word is processed, if neither the M nor the Σ criterion is reached within the response deadline specified by the T criterion, the target is classified as a “nonword”. Through these three adjustable decision criteria, M, Σ, and T, the model further explains the phenomenon described above: higher-frequency orthographic neighbours reach the activation threshold earlier and inhibit lower-frequency target words, thereby producing an inhibitory effect. However, because these models were developed primarily on the basis of findings from alphabetic languages, and because Chinese lacks a stable letter-level structure while neighbours sharing the first character are often more strongly semantically related, explanations based solely on orthographic similarity and within-level competition may not fully account for the consistently observed inhibitory neighbourhood frequency effect in Chinese.

### 1.3. ERP Evidence, Research Objectives, and Hypotheses

Neurolinguistic research has provided supportive evidence for the above proposal. For example, an interaction between the orthographic neighbourhood size (ONS) effect and word class has been observed in brain regions implicated in inhibitory control (Fiebach et al., 2007), and word class is also primarily associated with semantic processing (Zhang et al., 2011). Other researchers have proposed that inhibitory control helps readers avoid errors in which orthographically similar words are substituted for the target word (Christopher et al., 2012; Ahr et al., 2016; De Rom et al., 2023), while also suppressing lexical competition during lexical retrieval (Nozari, 2019). More recent evidence further suggests that difficulty suppressing competing orthographic representations is associated with increased neighbour-based errors during word reading (De Rom & Van Reybroeck, 2024). Research has suggested that, when competition among orthographic neighbours is strong during word reading, readers require stronger inhibitory mechanisms to suppress competing candidates and achieve correct lexical selection. That is, readers need to inhibit not only competition between the target word and its orthographic neighbours, but also competition among the orthographic neighbours themselves (Davis & Lupker, 2006). Lambon Ralph et al. (2017) proposed the Controlled Semantic Cognition framework (CSC), which highlights the role of inhibitory control within the semantic control network in regulating semantic processing. Neuroimaging evidence further supports the involvement of a distributed semantic-control network in semantic processing (Chiou et al., 2018; Jackson, 2021). Studies of patients with aphasia have likewise demonstrated a close relationship between semantic activation and inhibitory control (Biegler et al., 2008; Schnur et al., 2006; Wilshire & McCarthy, 2002). Evidence from research on reading disorders has similarly demonstrated the important role of inhibitory control in lexical-semantic activation and lexical selection (Mirman, 2011; Pestalozzi et al., 2018; Nozari, 2019; De Rom et al., 2023).

Existing evidence further indicates that individual differences in inhibitory control modulate orthographic neighbourhood effects in Chinese. In research on the orthographic neighbourhood size (ONS) effect, differences in inhibitory control were primarily reflected in the N400 rather than the P200, suggesting that its influence is more likely to occur at the stage of semantic activation than at the early stage of orthographic and phonological processing (Xiong et al., 2025). Despite these findings, an important gap remains. Previous studies of the ONF effect in Chinese have primarily focused on lexical characteristics and have rarely examined whether individual differences in cognitive control contribute to the effect. Although recent evidence suggests that inhibitory control can modulate orthographic neighbourhood size effects in Chinese, it remains unclear whether the same individual-difference factor also modulates the orthographic neighbourhood frequency effect. More importantly, ERP evidence is still lacking as to whether such modulation emerges during lexical-semantic processing, particularly in the N400 time window.

Researchers have used event-related potential (ERP) techniques to identify electroencephalographic (EEG) components associated with the ONF effect. For example, studies of alphabetic languages have found that target words with higher-frequency orthographic neighbours elicit larger N400 amplitudes (Chetail et al., 2012; Debruille, 1998; Laszlo & Federmeier, 2011; Molinaro et al., 2010). Evidence from Chinese reading similarly shows that target words with higher-frequency orthographic neighbours are associated with more negative N400 amplitudes (Huang et al., 2006). The N400 is a well-established electrophysiological index of lexical-semantic processing and is sensitive to the activation, retrieval, and integration of semantic information (Kutas & Federmeier, 2011; Jankowiak, 2021; Federmeier, 2022). Accordingly, this component was selected as a key electrophysiological index of the ONF effect.

However, some studies have found that the ONF effect is reflected not only in the N400 but also in the later LPC time window (Huang et al., 2006). The LPC refers to a late positive component that typically follows the N400 and has been associated with later semantic integration, lexical selection, and response-related processes (Huang et al., 2006; H. Liu et al., 2014, 2018; Schotter et al., 2023; Jiang et al., 2024). ERP studies of language control further suggest that inhibitory-control demands can be reflected in late positive activity during later stages of language processing (H. Liu et al., 2014, 2018; Jiang et al., 2024). Huang et al. (2006) provided electrophysiological evidence that the ONF effect in Chinese extends to the LPC time window, with larger LPC responses observed for target words accompanied by higher-frequency orthographic neighbours. The authors proposed that this later effect may reflect continued influences of neighbouring words on semantic integration and response selection. Orthographic neighbourhood effects may extend beyond the N400 into later positive activity, although the functional relationship between these effects remains uncertain (Holcomb et al., 2002). More recent ERP evidence has linked the LPC to later semantic integration during reading (Schotter et al., 2023). Similarly, the electrophysiological evidence reported by Li et al. (2017) and Huang et al. (2006) points to a relatively late locus of the ONF effect. Taken together, the LPC may represent another key index of the ONF effect.

Accordingly, the present study had two primary objectives. First, we sought to characterise the behavioural and electrophysiological signatures of the ONF effect during Chinese two-character word recognition. Second, we examined whether individual differences in inhibitory control modulate the ONF effect and whether such modulation is expressed primarily during later lexical-semantic processing, as indexed by the N400 and LPC components.

Based on these considerations, we hypothesised that inhibitory control interacts with the ONF effect during word recognition, and this interaction is primarily reflected at later stages of lexical processing. Specifically, individuals with LIC were expected to show longer reaction times, larger N400 amplitudes, and smaller LPC amplitudes when processing target words with higher-frequency orthographic neighbours. In contrast, individuals with HIC were expected to suppress the competitive activation of higher-frequency orthographic neighbours effectively and therefore show no significant differences in either behavioural measures or ERP components between conditions with and without higher-frequency orthographic neighbours.

## 2. Preliminary Experiment: Screening Individuals with Different Levels of Inhibitory Control

### 2.1. Method

#### 2.1.1. Participants

The sample comprised 211 university students (126 females, ages 18–25) recruited from Henan Normal University through campus advertisements. Participants were Mandarin-speaking Chinese adults and reported using Mandarin in daily communication. All individuals were right-handed and had normal reading abilities. Participants reporting a history of psychiatric disorders or previous participation in similar experiments were excluded. Before taking part in the study, participants signed informed consent forms and were paid after participation.

#### 2.1.2. Tasks

Flanker task: participants completed a Flanker task based on previously established experimental protocols (Cheng et al., 2021; Rey-Mermet et al., 2018). Each stimulus comprised five black arrows displayed in a horizontal row on a white background. Responses were based on the direction of the central arrow (left vs. right), while the four surrounding arrows provided task-irrelevant information. Congruent trials contained arrows pointing in a consistent direction, whereas incongruent trials involved a directional conflict between the central and surrounding arrows.

Stop-signal task: the stop-signal task was adapted from previous paradigms (Kleinsorge, 2021; Bissett et al., 2023). Each trial displayed a black “O” or “X” on a white background. Participants responded to the letter unless a red square appeared above it, in which case they were required to withhold their response.

#### 2.1.3. Procedure

The study took place in a quiet laboratory environment. Participants viewed stimuli on a computer monitor while maintaining a viewing distance of approximately 75 cm. Responses were made using a keyboard.

The Flanker task was administered first. The task consisted of 160 trials, including 80 congruent and 80 incongruent trials. Stimuli were presented in a fully randomised order, with a unique sequence generated for each participant. Each trial began with a centrally presented fixation cross (“+”) lasting 500 ms, followed by a 150 ms blank interval. A five-arrow array was then displayed for a maximum of 1500 ms. Participants were asked to identify the direction of the central arrow as quickly and accurately as possible by pressing the “F” key for left-pointing arrows and the “J” key for right-pointing arrows. The arrow array disappeared once a response was made, and a 500 ms blank interval was inserted before the next trial. Participants completed the task within approximately 7 min.

The stop-signal task was administered subsequently. This task also consisted of 160 trials, of which 25% were stop-signal trials (40 trials), with the remaining trials being standard response trials. Trials were presented in a random order for each participant. Each trial proceeded as follows: (1) a fixation cross (“+”) appeared centrally for 500 ms; (2) the fixation period was followed by a 150 ms blank interval; (3) a stimulus letter (“O” or “X”) was then presented for a maximum of 1500 ms. Participants were instructed to press the “F” key for “O” and the “J” key for “X”. When a red square appeared above the target letter as the stop signal, the ongoing response tendency had to be inhibited; and (4) a 500 ms blank interval followed either the participant’s response or stimulus offset. The task lasted approximately 7 min.

A practice phase preceded the formal experiment. Participants performed 12 practice trials in each task to become familiar with the experimental procedures. Entry into the formal session required an accuracy of at least 90% during practice; participants who did not meet this requirement continued practising until the criterion was achieved.

### 2.2. Screening Results

Reaction times (RTs) and accuracy rates were calculated separately for the two tasks, and RTs beyond ±3 SDs from the mean were excluded, accounting for 0.01% of all observations.

Behavioural performance in inhibitory-control tasks is commonly characterised using reaction time (RT) and accuracy (Schulz et al., 2023; Lee & Pitt, 2024). In the present study, accuracy was generally high across all participants (all above 90%), and no significant between-group differences were observed. Therefore, reaction time was used as the basis for group classification, with 36 participants selected for the HIC group and 36 for the LIC group. An extreme-group approach was adopted to obtain two clearly differentiated inhibitory-control groups for the subsequent ERP comparison. Following classification criteria used in previous studies (Feldt, 1961; Preacher et al., 2005), participants whose mean reaction times on both tasks fell within the fastest 27% of the overall sample were assigned to the HIC group, whereas those whose mean reaction times on both tasks fell within the slowest 27% were assigned to the LIC group. Reaction times for the two groups across both tasks are summarised in Table 1 as means and standard deviations, together with the corresponding independent-sample *t*-test results.

## 3. Formal Experiment: The Effect of Inhibitory Control on the ONF Effect

### 3.1. Method

#### 3.1.1. Participants

An a priori power analysis conducted using G*Power 3.1 (*f* = 0.30, *α* = 0.05, power = 0.95) yielded a target sample size of 66 participants. The 72 participants selected in the preliminary experiment took part in the formal experiment. After excluding participants with abnormal EEG recordings, the final sample comprised 68 valid participants (38 females, ages 18 to 25). The final sample comprised 34 participants in each inhibitory-control group.

#### 3.1.2. Experimental Design

A 2 × 2 mixed factorial design was employed. Inhibitory control was the between-subject factor (high vs. low), whereas ONF was the within-subject factor (high vs. low). Outcome measures included lexical decision reaction times and accuracy, as well as mean N400 and LPC amplitudes.

#### 3.1.3. Experimental Materials

Eighty sets of Chinese two-character words were selected from the Chinese Linguistic Data Consortium (2003) as experimental materials. Each set comprised two types of target words: one type contained higher-frequency orthographic neighbours (i.e., at least one orthographic neighbour had a higher word frequency than the target itself), whereas the other type contained no higher-frequency orthographic neighbours. The two target-word categories were matched on initial-character family size, word frequency, initial-character stroke count and frequency, as well as final-character family size, stroke count, and frequency, with no significant differences detected in any of these variables (all *p*s > 0.05). Pseudoword fillers were subsequently constructed by combining two characters selected from the corpus, with the resulting items matched to real words in terms of their constituent-character properties. The characteristics of all experimental materials are summarised in Table 2.

#### 3.1.4. Experimental Procedure

Data collection took place in a laboratory where ambient noise and lighting conditions were controlled. During the experiment, participants were seated approximately 75 cm from the monitor. The display settings, including resolution and refresh rate, were kept consistent with those used in the preliminary experiment.

Participants performed a lexical decision task consisting of 160 trials divided equally into two blocks. The order of stimuli within each block was randomised, with an equal number of trials included in both blocks. Each trial followed a fixed sequence: a fixation cross (“+”) was presented at the centre of the screen for 500 ms, followed by a 150 ms blank interval. A target word was subsequently displayed for 2000 ms, during which, participants were asked to judge whether it represented a real word or a pseudoword as quickly and accurately as possible. Responses were recorded by pressing the “J” key for real words and the “F” key for pseudowords. After a response was made, the target stimulus was removed, and a 150 ms blank interval preceded the next trial. Participants were given a short break between the two blocks and initiated the second block by pressing a key after the break. The task lasted approximately 15 min.

A practice phase comprising 12 trials preceded the formal task. They were required to achieve an accuracy level of 90% or above, before proceeding to the formal experiment.

### 3.2. Data Recording and Analysis

#### 3.2.1. Behavioural Data

Behavioural analyses were conducted on reaction times and accuracy. Trials involving incorrect responses or reaction times outside ±3 SDs of each participant’s mean were removed before statistical testing (0.05% of all trials). Two-way repeated-measure ANOVAs were performed in IBM SPSS Statistics 27.0, and experimental presentation and response collection were implemented using E-Prime 2.0.

#### 3.2.2. EEG Recording and Analysis

Continuous EEG was acquired using a 64-channel NeuroScan recording system (Compumedics Neuroscan, Charlotte, NC, USA) equipped with an Ag/AgCl electrode cap. Electrode placement followed the internationally standardised GB 10–20 system. CPz served as the online reference electrode during EEG recording, and the ground electrode (GND) was placed at the centre of the forehead. Vertical electrooculogram (VEOG) activity was recorded using electrodes positioned above and below the left eye, whereas horizontal electrooculogram (HEOG) activity was recorded using electrodes placed 1 cm lateral to the outer canthi of the left and right eyes. The online sampling rate was 1000 Hz, with a band-pass filter of 0.1–100 Hz. Electrode–scalp impedances were maintained below 5 kΩ.

EEG data were analysed offline using EEGLAB and ERPLAB in MATLAB R2022a, together with custom MATLAB scripts. Following re-referencing to the average of all electrodes, ocular artefacts were corrected using EEGLAB (Delorme & Makeig, 2004). The data were then band-pass filtered offline at 0.1–40 Hz and segmented into epochs from 200 ms before to 800 ms after stimulus onset, with the 200 ms prestimulus interval used for baseline correction. Independent component analysis (ICA) was subsequently applied to remove artefacts such as cardiac activity and eye blinks. Finally, trials with amplitudes exceeding ±75 μV were rejected, and only EEG data from trials with correct responses were included in the averaged waveforms. EEG analyses focused on the N400 (350–450 ms) and LPC (460–750 ms) components. Mean amplitudes of the N400 and LPC were analysed separately for correct-response trials in each experimental condition across all participants.

The scalp electrodes were divided into five regions: left anterior (F5, F3, F1, FC5, FC3, FC1), right anterior (F6, F4, F2, FC6, FC4, FC2), left posterior (CP5, CP3, CP1, P5, P3, P1), right posterior (CP6, CP4, CP2, P6, P4, P2), and central (FCz, C1, Cz, C2, CPz). A 2 (inhibitory control: high vs. low) × 2 (higher-frequency orthographic neighbours: present vs. absent) mixed design was employed. Repeated-measure analyses of variance (ANOVAs) were conducted separately for the behavioural data (reaction time and accuracy) and EEG data (mean amplitude of each ERP component). For the EEG data, separate repeated-measure ANOVAs were conducted on the N400 and LPC components at the electrode sites within each region. Only main effects and interactions reaching statistical or marginal significance are reported for the behavioural and EEG analyses. Violations of sphericity were corrected using the Greenhouse–Geisser procedure when required (Greenhouse & Geisser, 1959). Bonferroni correction was applied to follow-up pairwise and simple-effect comparisons where appropriate.

### 3.3. Results

#### 3.3.1. Behavioural Results

Descriptive statistics for reaction times and accuracy in the lexical decision task are summarised in Table 3.

(1) Reaction time

The repeated-measure ANOVA revealed a significant main effect of group, *F*(1, 66) = 16.30, *p* < 0.001, *η*^2^p = 0.20, with the HIC group showing shorter reaction times in the lexical decision task than the LIC group. The main effect of ONF was also significant, *F*(1, 66) = 7.81, *p* < 0.01, *η*^2^p = 0.11; responses were faster in the absence than in the presence of higher-frequency orthographic neighbours. However, the interaction between ONF and group did not reach significance (*p* = 0.71).

(2) Accuracy

Accuracy differed significantly between the two inhibitory-control groups *F*(1, 66) = 4.78, *p* < 0.05, *η*^2^p = 0.07, such that the HIC group was slightly less accurate than the LIC group. Accuracy was unaffected by ONF, and no evidence of an ONF-by-group interaction was found (all *p*s > 0.10).

#### 3.3.2. ERP Results

(1) N400 (350–450 ms)

**Figure 1:**
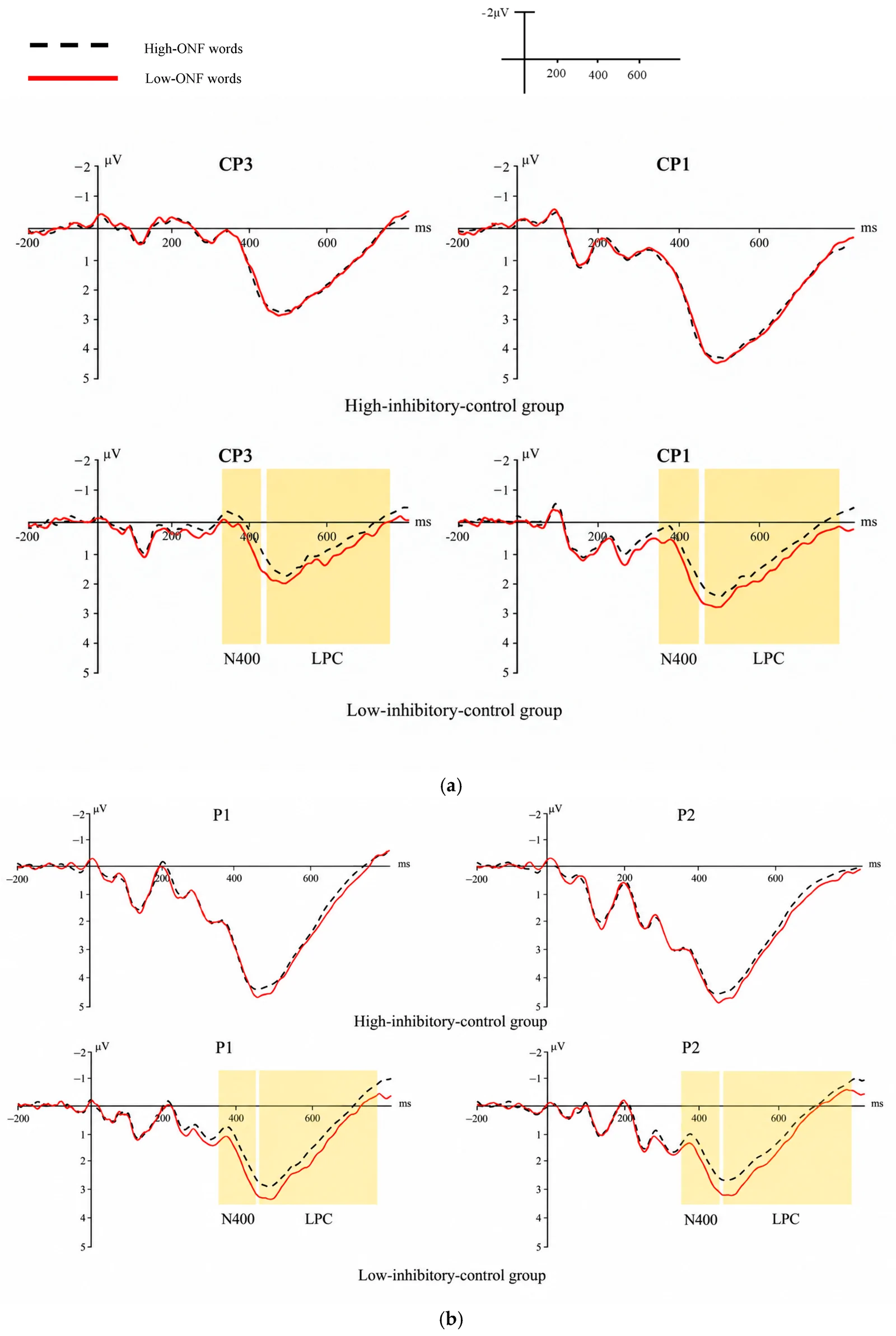
Grand-average N400 and LPC waveforms elicited by target words under different conditions. (**a**) N400 and LPC waveforms at representative electrodes in the left posterior region; (**b**) N400 and LPC waveforms at representative electrodes in the left and right posterior regions.

For each region, a 2 (group) × 2 (ONF) × 6 (electrode site) repeated-measure ANOVA was conducted on N400 amplitudes. In the left posterior region, N400 amplitudes differed significantly as a function of ONF (see Figure 1a), *F*(1, 66) = 5.97, *p* < 0.05, *η*^2^p = 0.08; amplitudes were larger when at least one higher-frequency orthographic neighbour was present than when no such neighbour was present. A significant interaction between ONF and group was observed, *F*(1, 66) = 5.34, *p* < 0.05, *η*^2^p = 0.08. Follow-up simple-effect analyses indicated no significant difference in N400 amplitudes between the two ONF conditions in the HIC group (*p* = 0.93). By contrast, among LIC participants, N400 amplitude increased when higher-frequency orthographic neighbours were present, relative to the condition in which such neighbours were absent, *F*(1, 66) = 11.30, *p* < 0.01, *η*^2^p = 0.15. The main effect of group was not significant (*p* = 0.19).

A similar pattern was observed in the right posterior region, where the main effect of ONF was marginally significant (see Figure 1b), *F*(1, 66) = 3.02, *p* = 0.09, *η*^2^p = 0.04. N400 amplitudes were larger for target words with higher-frequency orthographic neighbours than for those without. Group also showed a significant main effect, *F*(1, 66) = 7.56, *p* < 0.01, *η*^2^p = 0.10, with the LIC group exhibiting larger N400 amplitudes than the HIC group. No significant ONF × group interaction was observed (*p* = 0.14).

Analyses of the remaining regions showed that none of the other main effects or interactions reached significance (all *p*s > 0.10).

(2) LPC (460–750 ms)

A 2 (group) × 2 (ONF) × 6 (electrode site) repeated-measure ANOVA was conducted on LPC amplitudes. In the left posterior region (see Figure 1a), a significant ONF effect was observed, *F*(1, 66) = 5.86, *p* < 0.05, *η*^2^p = 0.08, such that LPC amplitudes were larger for target words without higher-frequency orthographic neighbours than for those with them. The ONF × group interaction was not significant (*p* = 0.78). However, further simple-effect analyses showed that, in the HIC group, LPC amplitudes did not differ significantly between the two ONF conditions (*p* = 0.14). Within the LIC group, LPC amplitudes differed marginally between the two ONF conditions (*p* = 0.06), with larger amplitudes elicited by target words without higher-frequency orthographic neighbours. The main effect of group did not reach significance (*p* = 0.17).

In the central region, a 2 (group) × 2 (ONF) × 5 (electrode site) repeated-measure ANOVA was conducted (see Figure 1a). A significant main effect of group was observed, *F*(1, 66) = 8.78, *p* < 0.01, *η*^2^p = 0.12, with larger LPC amplitudes in the HIC group than in the LIC group. No reliable ONF effect or ONF × group interaction was observed (all *p*s > 0.10).

Analyses of the remaining regions revealed no significant effects (all *ps* > 0.10).

**Figure 2:**
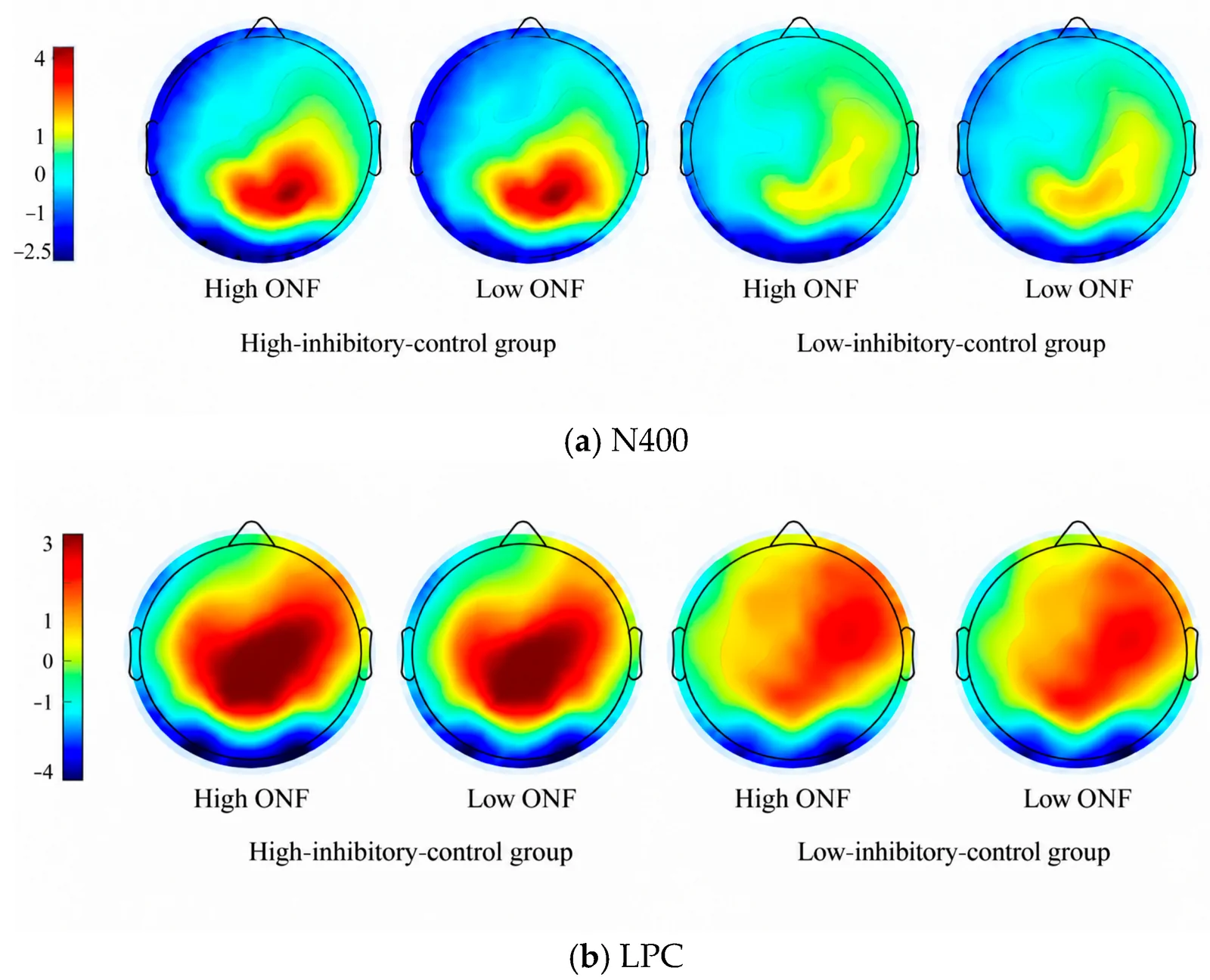
Scalp topographies of ERP activity in the N400 and LPC time windows.

The scalp topographies of the N400 and LPC components are shown in Figure 2.

## 4. Discussion

Using a lexical decision paradigm and EEG recordings, we examined the role of inhibitory control in the orthographic neighbourhood frequency effect during Chinese two-character word recognition The behavioural results showed that participants responded significantly faster to target words without higher-frequency orthographic neighbours than to those with higher-frequency orthographic neighbours, indicating an inhibitory effect of ONF on word recognition. However, no interaction was found between inhibitory control and the ONF effect. The EEG results showed that effects on the different components were distributed across different scalp regions. Across the left and right posterior regions, N400 amplitudes were larger for target words with higher-frequency orthographic neighbours than for those without. This pattern was also evident in the left posterior LPC component. Differences in ERP components elicited by the two ONF conditions were more pronounced in the LIC group, whereas these differences were less evident in the HIC group. These findings indicate that the ONF effect on target-word processing is modulated by individual differences in inhibitory control, and that this modulation is primarily reflected at relatively late stages of lexical processing.

### 4.1. ONF Effect in Chinese Two-Character Words

Behaviourally, target words with higher-frequency orthographic neighbours elicited longer response times, indicating an inhibitory ONF effect in Chinese two-character word recognition. This finding aligns with previous evidence from both alphabetic writing systems and Chinese (Grainger & Jacobs, 1996; Huang et al., 2006; Li et al., 2017; Sears et al., 2006; Warrington et al., 2018; Yao et al., 2022). According to the Interactive Activation Model and the Multiple Read-Out Model, orthographic neighbours that are similar to the target word but have a higher lexical frequency have higher levels of activation and are therefore more readily activated and able to compete with the target word, delaying its selection and recognition. We speculate that this may be because, in languages with relatively transparent orthographies, the correspondence between orthography and pronunciation is relatively consistent. Consequently, higher-frequency words with similar pronunciations are more likely to reach the M criterion (single-word activation) specified by the MROM and subsequently compete with lower-frequency target words across levels (Grainger & Jacobs, 1996). Compared with English, Chinese has a completely opaque orthographic system, and this account may therefore be insufficient to explain why inhibitory ONF effects have been consistently observed in lexical decision tasks in Chinese. The account proposed by Li et al. (2015) concerning the semantic stage of lexical processing may provide an explanation for this effect. Making a lexical decision involves more than the activation of individual characters; it requires the integration of character-level information into a coherent word representation. Therefore, lexical variables that emerge early during recognition may shape later semantic processing of the target word. Moreover, because higher-frequency words have higher levels of activation, higher-frequency orthographic neighbours that are activated earlier may inhibit lower-frequency target words.

In the formal experiment, the experimental manipulation involved whether target words had higher-frequency orthographic neighbours. In the higher-frequency neighbour condition, each target word had at least one orthographic neighbour that was more frequent than the target. The results showed clear N400 and LPC effects, both of which correspond to relatively late time windows of lexical processing. This finding provides further electrophysiological evidence that the ONF effect primarily influences the semantic processing stage of target-word recognition (Li et al., 2017). The EEG results further showed that, in both the left and right posterior regions, target words with higher-frequency orthographic neighbours elicited larger N400 amplitudes, consistent with previous findings (Huang et al., 2006). The N400 is closely associated with the retrieval and integration of lexical-semantic information (Kutas & Federmeier, 2011). During target-word recognition, higher-frequency orthographic neighbours are more readily activated and compete with the target word, thereby increasing the processing demands associated with semantic selection and confirmation of the target and eliciting a larger N400. This finding is consistent with the behavioural results, indicating that higher-frequency orthographic neighbours interfere with target-word processing.

The results further showed that, in the left posterior region, target words without higher-frequency orthographic neighbours elicited more positive LPC amplitudes. This finding differs from that of Huang et al. (2006), who found that target words with higher-frequency orthographic neighbours elicited more positive LPC amplitudes than those without higher-frequency orthographic neighbours in anterior and central brain regions. In the current study, a different pattern was observed in the left posterior region, with the electrodes showing significant differences located close to the top of the scalp. There may be three possible reasons for this. First, most of the target words selected in the present study were relatively low in frequency, and recognising these words may require readers to expend greater effort retrieving information from long-term memory. Second, the LPC effect may have overlapped with the N400 effect. Thus, target words with higher-frequency orthographic neighbours elicited larger N400 amplitudes, which may have resulted in more negative amplitudes at later stages. Third, the left posterior region is considered to be closely associated with the visual word form area (VWFA) (Hirshorn et al., 2016) and is sensitive to word frequency (Hauk et al., 2006). Therefore, changes in LPC amplitude over the left posterior region may more directly reflect lexical-level processing.

### 4.2. Differences in the ONF Effect Between Individuals with Different Levels of Inhibitory Control

The HIC group showed significantly shorter reaction times than the LIC group but slightly lower accuracy. This may reflect different speed–accuracy trade-offs, whereby the HIC group responded too quickly to the words and adopted a more “risky” strategy, resulting in errors on some trials. However, the ONF × group interaction was not significant, indicating that, at the behavioural level, no significant modulation of the ONF effect by inhibitory control was observed.

EEG analyses revealed a significant ONF × group interaction in the left posterior region. Specifically, the HIC group showed comparable N400 amplitudes across the two ONF conditions, whereas the LIC group showed larger N400 amplitudes for target words with higher-frequency orthographic neighbours. This pattern suggests that weaker inhibitory control may be associated with greater susceptibility to competing lexical-semantic activation during word recognition, thereby increasing processing demands during semantic selection and target-word confirmation. This interpretation is consistent with evidence that inhibitory deficits can contribute to semantic and lexical errors (Nozari, 2019) and that readers with weaker reading-related inhibitory control experience greater interference from orthographically similar words (Van Reybroeck & De Rom, 2020; De Rom & Van Reybroeck, 2024), and therefore, recruit more processing resources during the semantic selection and confirmation of the target word. The current results parallel the findings of Xiong et al. (2025), who demonstrated a more pronounced N400 neighbourhood effect in individuals with LIC during Chinese two-character word recognition. Evidence from aphasia research suggests that failures to suppress competing lexical representations may interfere with successful lexical retrieval (Nozari, 2019). Research on developmental dyslexia further suggests reading-specific difficulties in suppressing orthographic competition: children with dyslexia show reduced accuracy when orthographic neighbours interfere with target-word processing and make more neighbour-based guessing errors than typically developing readers (De Rom & Van Reybroeck, 2024; Van Reybroeck & De Rom, 2020). A recent meta-analysis of the N400 also showed reliable differences between individuals with dyslexia and typical readers in N400 effects during lexical-semantic processing (Basma et al., 2024). Overall, these findings suggest that individual differences in inhibitory control may influence ONF-related lexical-semantic processing.

Notably, the ONF × group interaction observed in the left posterior N400 was not evident in behavioural reaction times. This dissociation may reflect differences in the temporal sensitivity of the two measures. Whereas the N400 provides a temporally resolved index of lexical-semantic processing, reaction time reflects the aggregate outcome of multiple processing stages. Thus, inhibitory-control-related modulation of orthographic-neighbour competition may emerge during lexical-semantic processing without necessarily yielding a corresponding effect in overt behavioural performance.

For the LPC component, no significant interaction between ONF and group was observed in any brain region, indicating that the modulation of the ONF effect by inhibitory control was primarily reflected in the N400 stage and was not further supported at the LPC stage. However, the central region showed a significant main effect of group, with larger LPC amplitudes observed in the HIC group. This finding is consistent with previous reports that LPC amplitudes are predominantly distributed over parietal regions. During the later stages of word recognition, competition from higher-frequency orthographic neighbours may interfere with target-word processing. The larger LPC in the HIC group may reflect differences in later lexical selection and response-related control processes. Previous ERP studies have similarly linked individual differences in inhibitory control to LPC modulation during language control (H. Liu et al., 2014, 2018; Jiang et al., 2024). Previous studies suggest that inhibitory control may influence language processing at the lexical response-selection stage indexed by the LPC, with greater LPC modulation observed in the HIC group (H. Liu et al., 2014, 2018). Previous evidence indicates that differences in inhibitory control are reflected in language-switching costs observed in the LPC time window (Jiang et al., 2024).

When considered alongside the N400 findings in the left posterior region, the modulation of orthographic neighbourhood competition by inhibitory control may occur primarily during semantic activation and lexical selection. At the LPC stage, processing may involve more extensive re-evaluation of the target word, lexical selection, and response control. At this stage, the role of inhibitory control may shift from regulating competition from higher-frequency orthographic neighbours to controlling subsequent target-word selection and response processes. Consequently, individuals with different levels of inhibitory control may still show overall differences during later stages of processing. This may reflect a greater ability in individuals with HIC to suppress interference from competing words and to enhance familiarity with and confirmation of the target word.

## 5. Conclusions

We investigated the association between inhibitory control and the ONF effect during Chinese two-character word recognition. The behavioural results showed that higher-frequency orthographic neighbours inhibited target-word recognition. The ERP results further indicated that the modulation of the ONF effect by inhibitory control was primarily reflected at the stage of semantic activation. Individuals with LIC displayed a reliable ONF effect in the N400 time window, whereas individuals with HIC showed comparable neural responses across the two ONF conditions. Overall, these findings indicate that inhibitory control modulates the ONF effect in Chinese two-character word recognition, and may influence target-word recognition by limiting the competitive semantic activation of higher-frequency orthographic neighbours.

From an applied perspective, the findings suggest that Chinese word recognition is shaped by both lexical characteristics and individual differences in inhibitory control. Learners may differ in their susceptibility to interference from orthographically similar words. Future research should examine whether such differences can inform learner-sensitive approaches to Chinese vocabulary instruction.

## 6. Limitations and Future Directions

Methodological: the present study compared participants with high and low inhibitory control rather than directly manipulating inhibitory control, and therefore cannot establish a causal effect of inhibitory control on orthographic neighbourhood processing. In addition, the lexical decision task involves not only lexical-semantic processing but also decision and response processes, making it difficult to isolate the specific contribution of inhibitory control to lexical processing. Future studies could experimentally manipulate inhibitory-control demands and employ tasks that more directly index semantic processing to clarify its role in the ONF effect. The separate analyses of multiple scalp regions and ERP components may increase the overall risk of Type I error. Future studies should predefine primary time windows and regions of interest, apply appropriate corrections for multiple comparisons, and replicate key effects in independent samples (Luck & Gaspelin, 2017).

Sampling: the extreme-group approach excluded participants with intermediate levels of inhibitory control, limiting representation of the full ability continuum and potentially affecting effect-size estimates and generalisability (Preacher et al., 2005; Fisher et al., 2020). Future studies should treat inhibitory control as a continuous variable and examine its relationship with ONF-related behavioural and ERP measures.

Stimulus-material: because character-sharing words in Chinese may also share morphological or semantic information, this factor may have contributed to the observed effects. Future studies should assess and control semantic relatedness across ONF conditions.

Theoretical: the present findings suggest that inhibitory control is associated with ONF-related lexical-semantic processing, but the underlying mechanism remains unclear. The N400 effect cannot determine whether inhibitory control reflects direct suppression of competing orthographic neighbours or broader lexical-semantic control. Future studies should manipulate lexical competition more directly to distinguish these possibilities.

## Figures and Tables

**Table 1:** Mean reaction times by inhibitory-control group and task.

Group/Statistic	Flanker	Stop-Signal
H	430.00 (24.67)	462.13 (29.76)
L	542.59 (50.46)	579.63 (41.53)
*t*(70)	−12.027	−13.799
*p*	<0.001	<0.001

Note. H *=* HIC group, L = LIC group.

**Table 2:** Stimulus characteristics.

	High ONF [Mean (SD)]	Low ONF [Mean (SD)]	*p*
Initial ONS	31.58 (19.40)	31.01 (19.38)	0.93
Initial SN	8.44 (2.44)	8.75 (2.31)	0.41
Initial CF	2.31 (0.59)	2.28 (0.58)	0.76
Final ONS	61.01 (49.24)	58.25 (46.79)	0.72
Final SN	7.63 (2.59)	7.26 (2.25)	0.35
Final CF	2.79 (0.59)	2.80 (0.59)	0.96
WF	1.11 (0.50)	1.16 (0.49)	0.50

Note. ONS = orthographic neighbourhood size; SN = stroke number; CF = character frequency; WF = word frequency; High ONF = target words with at least one higher-frequency orthographic neighbour; Low ONF = target words without higher-frequency orthographic neighbours.

**Table 3:** Lexical decision performance (reaction times and accuracy) by inhibitory-control group.

Group	High ONF		Low ONF	
	RTs	Accuracy	RTs	Accuracy
*H*	570.36 (44.16)	0.94 (0.04)	566.02 (41.22)	0.94 (0.04)
*L*	628.70 (71.35)	0.96 (0.03)	623.04 (73.18)	0.96 (0.03)

H = HIC group, L = LIC group.

## Data Availability

The behavioural and EEG data supporting the findings of this study are available from the corresponding author upon reasonable request.
